# Relationship between illness severity scores in the ICU

**DOI:** 10.1186/cc11013

**Published:** 2012-03-20

**Authors:** A Schneider, M Lipcsey, M Bailey, D Pilcher, R Bellomo

**Affiliations:** 1 Austin Health, Heidelberg, Australia; 2 Monash University, Melbourne, Australia; 3 ANZICS, Melbourne, Australia

## Introduction

Many different illness severity scores are used to report the estimated risk of death (ROD) of patients in clinical research. Such variability makes mortality comparison between studies difficult. Accordingly, it would be desirable to establish a methodology to translate the value obtained from one scoring system into an estimated equivalent value for another scoring system.

## Methods

We used the adult patient database of the Australian and New Zealand (ANZ) Intensive Care Society to obtain simultaneous APACHE II (APII), APACHE III (APIII) and SAPS II scores. We used linear regression analyses to create models enabling translation of one score into another. These analyses were performed for the whole cohort, after exclusion of cardiac surgery patients and after matching for similar risk of death.

## Results

We obtained complete data for three illness severity scores (SAPS II, APII, and APIII) in 636,431 admissions. There was a good correlation between the APIII and APII scores (*r*^2 ^= 0.76). The overall model was APIII = 3.09 × APII + 5.8. The APIII/APII coefficient (SE) was 3.09 (0.002) for the whole cohort, 3.1 (0.002) after exclusion of cardiac surgery patients and 2.98 (0.01) after exclusion of patients with an absolute difference in ROD >1% between the two scores. There was a similar correlation between the APIII and the SAPS II scores (*r*^2 ^= 0.76). The overall model was APIII = 1.47 × SAPS II + 8.6. The APIII/SAPS II coefficient (SE) was 1.47 (0.001) for the whole cohort, 1.49 (0.001) after exclusion of cardiac surgery patients and 1.55 (0.006) after exclusion of patients with an absolute difference in ROD >1% between the two scores. Finally, the correlation between the APII and SAPS II scores was moderate (*r*^2 ^= 0.63). The overall model was APII = 0.36 × SAPS II + 4.4. The APII/SAPS II coefficient (SE) was 0.36 (0.0003) for the whole cohort, 0.37 (0.0004) after exclusion of cardiac surgery patients and 0.39 (0.002) after exclusion of patients with an absolute difference in ROD >1%.

## Conclusion

Simple and robust translational formulas can be developed to allow clinicians to compare illness severity in intensive care studies of similar patients when such illness severity is expressed with different scoring systems.

